# Supplementary material for: Protective factors, risk factors, and intervention strategies in the prevention and reduction of crime among adolescents and young adults aged 12–24 years: A scoping review protocol
Source: PLoS One. 2024 Nov 19;19(11):e0312684. doi: 10.1371/journal.pone.0312684 (PMC11575785; doi:10.1371/journal.pone.0312684)
Supplement: Attachment — Submitted filename: REVIEWERS COMMENTS.pdf [file pone.0312684.s004.pdf]

Thank for the opportunity to review this manuscript. The manuscript was written according to the journal's guidelines. However, several issues have been identified, as outlined below:

(i) Title:

I suggest that the title be revised to include the phrase "adolescent and young adults aged 12-24 years old" as follows:

Protective factors, risk factors, and intervention Strategies in the prevention and reduction of crime among adolescents and young adults aged 12-24 years: A scoping review protocol.

(ii) Manuscript Formatting Guidelines:

All headings should be written in sentence case (capitalize only the first word of the heading, the first word of the subheading, and any proper nouns).

1. ABSTRACT

The use of future tense and "key" concepts

1.1. Main aim of the study:

"The main objective of the study is to collate key risk/protective factors and intervention strategies in identifying youth at risk of crime."

The aim of the study could be more precisely stated. Instead of just mentioning *"collate"* it should emphasize the purpose behind collating these factors and strategies.

Example: The aim of this scoping review is to identify and map the key risk and protective factors……

1.2. Results:

The abstract currently does not include a summary of the results, which is a crucial component of a comprehensive abstract. This omission makes it challenging for readers to understand the primary findings and their significance.

Although the scoping review protocol itself may not have results yet, the abstract should mention the anticipated outcomes, or the kind of data expected to be synthesized. This helps set expectations and provides a clearer picture of the review's potential impact.

Consult: http://dx.doi.org/10.4103/ijamr.ijamr_91_23

1.3. Use of Future Tense:

The use of future tense (“will be used,” “will be searched,” “will include”) is inappropriate for a scoping review protocol that has already been completed. The use of this tense might confuse readers about the current stage of the study.

1.4. Use of “Key” Term:
“Current reviews in the area do not include literature discussing ***key*** at risk populations such as First Nations groups from countries such as Australia, Canada and New Zealand.” The use of the term “key” without clear context can be vague.

2. INTRODUCTION

2.1. Context and significance:

“Crime among young people remains a pressing issue globally, with a particular emphasis on drug-related offenses such as possession and trafficking. While there has been a general decline in youth offending over the past three decades, especially in property-related and violent crimes, regional variations persist [4].”

Add specific statistics and trends from authoritative sources like the UNODC to underscore the global significance of the issue. For example: https://dataunodc.un.org/

2.2. Problem definition and significance.

The introduction briefly defines the problem but does not thoroughly explain why it is critically important.

Suggested Source: A source like the “World Health Organization (WHO) Report on Youth and Crime” might also provide insights into the health impacts of youth crimes.

2.3. Relevant controversies or disagreements

Discuss some of the ongoing debates or controversies regarding the effectiveness of various intervention strategies.

For instance, highlight arguments surrounding rehabilitation and therapeutic interventions. See: https://doi.org/10.1037/tra0001073 and https://doi.org/10.1007/s12207-024-09497-5

2.4. Overall Aim:

I suggest that you conclude with a clear statement of the rationale and aim of study. This is often beneficial for a seamless transition from providing background information to stating the rationale and aim of the study. This approach ensures that readers understand the context and significance of the research from the outset along with the

rationale and aim within the introduction itself without having readers to navigate through separate subsections.

3. METERIAL AND METHODS

3.1. Preregistration of protocols

While the authors mention preregistration with the Open Science Framework, it does not provide an open-access direct link or detailed information about the preregistered protocol. This reduces transparency and makes it difficult for readers to access the full details of the review protocol.

3.2. Consistency in Citation Style (Vancouver Style)

Page 4, *"The scoping review method was selected as it is useful for identifying gaps in, and the range of literature in an area (Munn et al., 2018)."*

The provided text above introduces a mix of citation styles. Consistency in citation style is crucial for clarity and readability throughout manuscript.

3.3. Step 1: Identification of the Research Questions

Page 5 *"Which intervention strategies are promising to reduce and prevent the risk of crime in 12-24-year-old youth?"*

The use of the phrase *"12-24-year-old youth"* limits clarity and specificity, here is a suggested and commonly accepted phrase in a professional and academic context ***"adolescents and young adults aged 12 to 24".***

Question 3: "What is the extent, range, and nature of the evidence regarding crime reduction and prevention strategies in 12-24-year-olds?"

Question 4: "Which intervention strategies are promising to reduce and prevent the risk of crime in 12-24-year-old youth?"

While the overarching goal of both questions is to inform the development of effective crime prevention strategies for adolescents and young adults, it is thus essential to ensure that these questions complement each other and do not unnecessarily duplicate efforts during the scoping review process. These questions are similar in terms of objective, scope, and implications, suggesting redundancy.

3.4. Eligibility Criteria:

Add detailed explanations or references for key terms and concepts to ensure consistency and replicability. For example, provide legal definitions for different types of crimes mentioned, and elaborate on what constitutes “at risk of offending.”

In the context of the scoping review, the term “general population” typically refers to individuals who are not specifically targeted or categorized based on particular characteristics or conditions.

Provide clarity on the meaning of “general population” within the specified context of the review on the following aspect mentioned **on page 6**.

b) Universal interventions in the ***general population***

c) Prevention/early intervention in the ***general population***

Page 7, “Grey and unpublished literature” These terms mean the same thing, authors should decide on which one they’d prefer to employ.

Page 7, “Two reviewers will engage in the data charting process. The data items recommended by the JBI Manual (Aromataris &Munn, 2020).”

Authors must be consistent with the recommended citation style (Vancouver style) through the manuscript.

3.5. Data items

Specific data that will be extracted from eligible studies include:

“a) Citation details (author/s, title, date of publication, **<u>country</u>**)

“b) **Country**”

These two items will be duplicates.

“f) Exposure and outcome assessment”

Exposure and outcome assessment aligns with the PICO (Population, Intervention, Comparison, Outcome) framework rather than the PCC (Population, Concept, Context) framework adopted by this study. Given that a scoping review utilises the PCC framework, authors may need to carefully consider revising this.

3.6. Flow diagram.

The authors mention the inclusion of a flow diagram but lacks specific details. It is essential to specify the stages that will be detailed in the flow diagram, such as the

number of records identified, screened, eligible, and included in the review. According to PRISMA-ScR guidelines, the flow diagram should be comprehensive.

3.7. Critical Appraisal and Justification

Page 8, "No risk of bias or quality assessment will be undertaken on included sources, as is outlined in the Arksey & O'Malley framework (2005)."

While the justification for not conducting a risk of bias assessment is provided, it is beneficial to add a more detailed explanation. This could include the rationale behind focusing on breadth rather than depth and how this approach aligns with the objectives of a scoping review.

4. DISCUSSION

4.1. Synthesis of findings

The discussion does not provide a detailed synthesis of the findings from the scoping review.

I strongly suggest that authors integrate specific findings from the review to illustrate key points. For instance, mention specific risk and protective factors identified and any intervention strategies that showed promise.

See. https://onlinelibrary.wiley.com/doi/book/10.1002/9780470693926

5. References

From page 13 – 14: *The following references have not been formatted according to the numerical system required by the Vancouver style.*

Tricco, A. C., Lillie, E., Zarin, W., O'Brien, K. K., Colquhoun, H., Levac, D., Moher, D., Peters, M. D. J., Horsley, T., Weeks, L., Hempel, S., Akl, E. A., Chang, C., McGowan, J., Stewart, L., Hartling, L., Aldcroft, A., Wilson, M. G., Garritty, C., Lewin, S., Godfrey, C. M., Macdonald, M. T., Langlois, E. V., Soares-Weiser, K., 13 Moriarty, J., Clifford, T., Tunçalp, Ö., & Straus, S. E. (2018). PRISMA Extension for Scoping Reviews (PRISMA-ScR): Checklist and Explanation. Ann Intern Med, 169(7), 467-473. https://doi.org/10.7326/m18-0850

Aromataris E, Munn Z (Editors). JBI Manual for Evidence Synthesis. JBI, 2020. Available from https://synthesismanual.jbi.global. https://doi.org/10.46658/JBIMES-20-01

Levac, D., Colquhoun, H., & O'Brien, K. K. Scoping studies: Advancing the methodology. Implementation Science 2010, 5, 69. doi: 10.1186/1748-5908-5-69.

Thank you.
