# Supplementary material for: Protective factors, risk factors, and intervention strategies in the prevention and reduction of crime among adolescents and young adults aged 12–24 years: A scoping review protocol
Source: PLoS One. 2024 Nov 19;19(11):e0312684. doi: 10.1371/journal.pone.0312684 (PMC11575785; doi:10.1371/journal.pone.0312684)
Supplement: Attachment — Submitted filename: Point-by-point response factors 11.09.2024.pdf [file pone.0312684.s005.pdf]

Point-by-point responses for comments

Dear Professor AKM Alamgir, PhD, Academic Editor, PLOS ONE

We would like to thank you very much for giving us the opportunity to re-revise our manuscript entitled "***Protective factors, risk factors, and intervention Strategies in the prevention and reduction of crime among adolescents and young adults aged 12-24 years: A scoping review protocol***" **[PONE-D-23-15234R1] - [EMID:3381d626f3677521].** We would also like to express our thanks to the reviewers for sharing their expertise, detailed reviews, and providing us with valuable suggestions and constructive comments on our manuscript. We have fully addressed their comments in the revised manuscript. Following each comment and suggestion, the answer is provided together with references to the page and lines where the changes were made.

We believe changes have resulted in a significantly improved manuscript, which you will find uploaded alongside this document.

Editor and Reviewers' comments:

Editor's comments:

Please provide a point-by-point response to the reviewers' comments

Response: *As requested, we have included a point-by-point response to all reviewer comments in this document.*

Reviewer 3

General comments

Thank for the opportunity to review this manuscript. The manuscript was written according to the journal's guidelines. However, several issues have been identified, as outlined below:

(i)**Q1. Title:**

I suggest that the title be revised to include the phrase "adolescent and young adults aged 12-24 years old" as follows: Protective factors, risk factors, and intervention Strategies in the prevention and reduction of crime among adolescents and young adults aged 12-24 years: A scoping review protocol.

Author reply: Thank you for your suggestion. We have now revised the title as recommended: *Protective factors, risk factors, and intervention strategies in the prevention and reduction of crime among adolescents and young adults aged 12-24 years: A scoping review protocol."* **(See pages 1, title page, lines 1-4):**

Q2. ***Manuscript Formatting Guidelines:*** *All headings should be written in sentence case (capitalize only the first word of the heading, the first word of the subheading, and any proper nouns).*

Author's Reply: *Thank you for the reminder. We have updated all headings in the manuscript to follow the sentence case formatting as per the guidelines, capitalizing only the first word of each heading, the first word of each subheading, and any proper nouns.*

1. ***ABSTRACT***

The use of future tense and "key" concepts

1.1. Main aim of the study: *"The main objective of the study is to collate key risk/protective factors and intervention strategies in identifying youth at risk of crime." The aim of the study could be more precisely stated. Instead of just mentioning "collate" it should emphasize the purpose behind collating these factors and strategies. Example: The aim of this scoping review is to identify and map the key risk and protective factors……*

Author's Reply: Thank you. We have revised the aim as follows *"The aim of this scoping review is to identify and map the key risk and protective factors, along with intervention strategies, that are essential for recognizing adolescents and young adults at risk of crime."* **(See pages 2, title page, lines 50-52):**

1.2. Results: *The abstract currently does not include a summary of the results, which is a crucial component of a comprehensive abstract. This omission makes it challenging for readers to understand the primary findings and their significance. Although the scoping review*

protocol itself may not have results yet, the abstract should mention the anticipated outcomes, or the kind of data expected to be synthesized. This helps set expectations and provides a clearer picture of the review's potential impact. Consult: ***http://dx.doi.org/10.4103/ijamr.ijamr_91_23***

Author's Reply: Thank you. We have revised the conclusion section to include key anticipated results as follows *"**Conclusion:** This scoping review protocol aims to systematically map key risk and protective factors, as well as intervention strategies, for preventing and reducing youth crime, including among First Nations populations. Anticipated findings suggest that current research has extensively examined factors across all levels of the socioecological model—from individual to community levels—revealing a predominant focus on individual-level predictors such as substance use, prior criminal history, and moral development. The review is expected to identify effective interventions that address critical factors within each domain, including Multisystemic Therapy (MST) and Multidimensional Treatment Foster Care (MTFC), which have shown promise in reducing youth crime. Additionally, it will likely highlight significant trends in risk and protective factors, such as the dual role of academic achievement—both as a risk and protective factor—and the impact of family-based interventions. The review will also address gaps in research, particularly regarding Indigenous youth, underscoring the need for targeted studies to better understand their unique challenges. These findings will guide future research and inform the development of comprehensive prevention and early intervention programs tailored to diverse youth populations.".* **See pages 2-3, abstract section, lines 63-78):**

1.3. Use of Future Tense:

The use of future tense ("will be used," "will be searched," "will include") is inappropriate for a scoping review protocol that has already been completed. The use of this tense might confuse readers about the current stage of the study

Author's Reply: This has been fixed now.

1.4. Use of "Key" Term:

"Current reviews in the area do not include literature discussing key at risk populations such as First Nations groups from countries such as Australia, Canada and New Zealand."

The use of the term "key" without clear context can be vague.

Author's Reply: This has been fixed now.

2. INTRODUCTION

2.1. Context and significance:

"Crime among young people remains a pressing issue globally, with a particular emphasis on drug-related offenses such as possession and trafficking. While there has been a general decline in youth offending over the past three decades, especially in property-related and violent crimes, regional variations persist [4]."Add specific statistics and trends from authoritative sources like the UNODC to underscore the global significance of the issue. ***For example*** ***https://dataunodc.un.org/***

Author's Reply: Thank you for raising these important concerns. We have revised the introduction section to include the statistics as follows: "Introduction:

Crime among young people remains a pressing issue globally, with a particular emphasis on drug-related offenses such as possession and trafficking. While there has been a general decline in youth offending over the past three decades, especially in property-related and violent crimes, regional variations persist [1, 2]. For instance, in the United States, the number of violent crime arrests involving youth reached a new low by 2020, representing a 78 percent decrease from the 1994 peak and a 56 percent decline between 2010 and 2020 [2]. Despite this general decline, drug-related offenses have been increasing in certain regions, such as

Europe. From 1990 to 2000, Western Europe saw a notable rise in drug offenses, correlated with the increased availability of drugs in European markets and the rise of drug use as shown by other indicators [1].” **See page 3, introduction section, lines 79-88):**

2.2. Problem definition and significance.

The introduction briefly defines the problem but does not thoroughly explain why it is critically important.

Suggested Source: A source like the “World Health Organization (WHO) Report on Youth and Crime” might also provide insights into the health impacts of youth crimes.

Author's Reply: We have revised the introduction section to including significance of the study as follows: *Youth involvement in criminal behavior carries severe physical, mental, and social health consequences, underscoring the urgent need for effective prevention and early intervention strategies. The health impacts of youth crime are profound: young offenders and victims often experience severe mental health issues, including increased rates of depression, anxiety, and trauma, which can have lasting effects on their development and overall well-being [3]. These mental health challenges are compounded by physical health problems such as injuries sustained during criminal activities and long-term health complications associated with substance abuse [4]. The link between drug use and criminal behaviour not only exacerbates these health issues but also perpetuates a cycle of addiction and offending.*

The significance of addressing youth crime extends beyond individual health concerns to broader societal impacts. Families and communities face heightened healthcare costs and diminished quality of life due to the repercussions of youth offending. Communities experience reduced safety, increased fear, and economic costs associated with criminal justice responses. Risk factors contributing to youth offending are varied and include family dysfunction, exposure to abuse or community violence, substance misuse, and socioeconomic disparities

[16-20]. hese factors highlight the complexity of the issue and the need for targeted prevention strategies that address both the immediate and underlying causes of youth criminal behaviour.

Understanding these severe consequences and diverse risk factors is crucial for developing comprehensive approaches to prevent youth crime and mitigate its impact on individuals and communities [3, 4]. **See pages 3-4, introduction section, lines 94-113):**

3.Relevant controversies or disagreements: *Discuss some of the ongoing debates or controversies regarding the effectiveness of various intervention strategies.*

For instance, highlight arguments surrounding rehabilitation and therapeutic interventions. ***See: https://doi.org/10.1037/tra0001073 and https://doi.org/10.1007/s12207-024-09497-5***

Author's Reply: Thank you for raising these important concerns. We have now discussed some of the ongoing debates or controversies regarding the effectiveness of various intervention strategies. See the statement included in the introduction section **[See page 5, introduction section, lines 125-142)]**: *Effective intervention strategies for youth crime encompass both therapeutic and non-therapeutic approaches. Therapeutic interventions like cognitive behavioural therapy (CBT) and dialectical behavioural therapy (DBT) have shown promise in addressing risk-taking behaviours and reducing offending, yet there remains debate about their overall effectiveness and applicability to diverse populations[5].. Critics argue that while these therapies can be beneficial, they may not adequately address broader socio-economic and systemic factors contributing to criminal behaviour [6]. For instance, the efficacy of CBT and DBT can vary significantly based on individual differences and the quality of implementation, raising concerns about their universal applicability. Additionally, Functional Family Therapy (FFT), which offers a holistic approach by addressing familial dynamics and employing a multi-systemic and trauma-informed perspective, is praised for its comprehensive approach. However, challenges such as engaging all family members and*

maintaining consistent implementation can impact its success [5]. The debate also extends to the need for culturally safe practices, peer support, and flexible therapeutic approaches, which are crucial for effective interventions with at-risk youth but can be challenging to implement consistently [6]. These ongoing controversies highlight the complexities and varied outcomes associated with different therapeutic strategies, underscoring the need for continued evaluation and adaptation of interventions.

2.4. Overall Aim:

I suggest that you conclude with a clear statement of the rationale and aim of study. This is often beneficial for a seamless transition from providing background information to stating the rationale and aim of the study. This approach ensures that readers understand the context and significance of the research from the outset along with the

rationale and aim within the introduction itself without having readers to navigate through separate subsections.

Author's Reply: We have revised the introduction section to include the rationale and aim of study follows: "***Aim and significance of study***

Our preliminary review of the existing literature reveals a critical gap in meta-analyses or umbrella reviews specifically addressing risk and protective factors, as well as prevention strategies for youth crime, particularly for First Nations people who are disproportionately incarcerated [40]. The existing research predominantly focuses on broad or non-specific populations, leaving a significant void in targeted analyses for marginalized groups. Identifying robust risk and protective factors is essential for developing precise predictive tools and effective intervention strategies. This scoping review aims to address this gap by systematically mapping the key risk and protective factors, as well as the intervention strategies, to prevent and reduce crime among youth aged 12-24 who are at risk of offending.

The significance of this study lies in its potential to provide a comprehensive overview of factors influencing youth crime, tailored to the unique needs of First Nations communities and other high-risk groups. By consolidating and synthesizing existing research, this review will offer critical insights that can guide the development of targeted prevention and early intervention programs. The findings are expected to inform policy, improve program design, and contribute to reducing youth crime rates. Ultimately, this study will establish a benchmark for future research, helping to bridge existing gaps and foster more effective and culturally relevant interventions". **See pages 5-6, method section, lines 143-160):**

3.METERIAL AND METHODS 3.1.

Preregistration of protocols

While the authors mention preregistration with the Open Science Framework, it does not provide an open-access direct link or detailed information about the preregistered protocol. This reduces transparency and makes it difficult for readers to access the full details of the review protocol.

Author's Reply: *We have now updated the link*: *https://osf.io/vka3p*. **See page 7, method section, line 169).**

2. Consistency in Citation Style (Vancouver Style)

Page 4, "The scoping review method was selected as it is useful for identifying gaps in, and the range of literature in an area (Munn et al., 2018)."

The provided text above introduces a mix of citation styles. Consistency in citation style is crucial for clarity and readability throughout manuscript.

Author's Reply: *We have updated the citation according to the PLOS ONE's guidelines and ensured consistency throughout the manuscript. Thank you for bringing this to our attention.*

3.3. Step 1: Identification of the Research Questions

Page 5 "Which intervention strategies are promising to reduce and prevent the risk of crime in 12-24-year-old youth?"

The use of the phrase "12-24-year-old youth" limits clarity and specificity, here is a suggested and commonly accepted phrase in a professional and academic context "adolescents and young adults aged 12 to 24".

Question 3: "What is the extent, range, and nature of the evidence regarding crime reduction and prevention strategies in 12-24-year-olds?"

Question 4: "Which intervention strategies are promising to reduce and prevent the risk of crime in 12-24-year-old youth?"

While the overarching goal of both questions is to inform the development of effective crime prevention strategies for adolescents and young adults, it is thus essential to ensure that these questions complement each other and do not unnecessarily duplicate efforts during the scoping review process. These questions are similar in terms of objective, scope, and implications, suggesting redundancy.

Author's Reply: *Thank you for your valuable feedback. This has now been addressed and revised accordingly.* **(See page 7, method section, lines 178-183).**

3.4. Eligibility Criteria:

Add detailed explanations or references for key terms and concepts to ensure consistency and replicability. For example, provide legal definitions for different types of crimes mentioned, and elaborate on what constitutes "at risk of offending."

In the context of the scoping review, the term "general population" typically refers to individuals who are not specifically targeted or categorized based on particular characteristics or conditions.

Provide clarity on the meaning of "general population" within the specified context of the review on the following aspect mentioned on page 6.

b) Universal interventions in the general population

c) Prevention/early intervention in the general population):

Author's Reply: *Thank you for your valuable feedback. This has now been addressed and revised accordingly. See below the corrections:*

a) Global Context: Studies conducted across various countries and regions to provide a broad understanding of the issue.

b) Universal Interventions in the General Population: Interventions aimed at the entire population, not limited by specific characteristics, conditions, or risk factors. The term "general population" refers to individuals who are not specifically categorized by certain risk factors or conditions.

c) Prevention/Early Intervention in the General Population: Programs and strategies designed to prevent the onset of criminal behavior or address early signs of such behavior within the general population.

d) Targeted Interventions in Clinical and Population-Based Settings: Interventions specifically aimed at high-risk groups or those already engaged in offending behavior, within both clinical settings (e.g., mental health services) and broader community-based contexts.

e) Interventions for First Nations Populations: Studies focused on interventions specifically designed for Indigenous communities, recognizing the unique cultural and social contexts of these groups.

f) Grey Literature: Inclusion of non-peer-reviewed sources, such as reports, theses, and government publications, to capture a wider range of evidence.

g) No Date Restriction: Studies from any publication year were eligible, allowing for a comprehensive review of the literature

h) Language: Only studies published in English were considered, to ensure accessibility and comprehension. **(See pages 8-9, method section, lines 203-223).**

***Page** 7, "Grey and unpublished literature" These terms mean the same thing, authors should decide on which one they'd prefer to employ.*

Author's Reply: This has been fixed now.

***Page** 7, "Two reviewers will engage in the data charting process. The data items recommended by the JBI Manual (Aromataris &Munn, 2020)."*

Authors must be consistent with the recommended citation style (Vancouver style)

Author's Reply: This has been fixed now.

("f) Exposure and outcome assessment"

Exposure and outcome assessment aligns with the PICO (Population, Intervention, Comparison, Outcome) framework rather than the PCC (Population, Concept, Context) framework adopted by this study. Given that a scoping review utilises the PCC framework, authors may need to carefully consider revising this): ***f) Exposure and outcome assessment***

Author's Reply: *This concern has now been addressed and revised accordingly*. **(See pages 10-11, method section, lines 255-271).** We have added the following in the revised version:

"In the context of this scoping review, the exposure and outcome assessment process should align with the PCC (Population, Concept, Context) framework rather than the PICO (Population, Intervention, Comparison, Outcome) framework, which is typically used for systematic reviews and meta-analyses. The PCC framework is more suitable for scoping reviews as it allows for broader exploration of the available literature without the strict comparison of interventions required by PICO.

Under the PCC framework, exposure is defined in terms of the specific characteristics or conditions within the population of interest (e.g., demographic factors, socio-economic status, or environmental factors) that may influence the risk of engaging in criminal behavior. Outcomes refer to the range of consequences or effects observed within the population, including rates of offending, recidivism, or the success of intervention strategies.

Given the nature of a scoping review, the emphasis is placed on mapping and summarizing the existing evidence, identifying gaps in the literature, and understanding the context in which these exposures and outcomes are observed. Therefore, authors should ensure that the assessment process remains consistent with the PCC framework by focusing on the conceptual relationships and broader context of the findings rather than strictly comparing interventions and their outcomes".

3.6. Flow diagram *: The authors mention the inclusion of a flow diagram but lacks specific details. It is essential to specify the stages that will be detailed in the flow diagram, such as the number of records identified, screened, eligible, and included in the review. According to PRISMA-ScR guidelines, the flow diagram should be comprehensive.*

***Author's Reply*:** Thank you for your valuable feedback. This has now been addressed and revised accordingly. **We also included this description in the method section "**In our

preliminary systematic search of electronic databases and manual search of evidence, we found 9,769 sources, of which 235 met the predefined criteria (**Fig 1**)". (**See pages 11, method section, lines 282-284 and page 19 line 475-478**):

3.7. Critical Appraisal and Justification

Page 8, "No risk of bias or quality assessment will be undertaken on included sources, as is outlined in the Arksey & O'Malley framework (2005

Author's Reply: Thank you for raising these important concerns. We have revised this section as follows: "Critical appraisal of individual sources of evidence

"In accordance with the Arksey and O'Malley framework (2005) (2005) [42]. the scoping review does not involve a formal risk of bias or quality assessment of included sources. This approach is justified by the specific objectives of a scoping review, which are to map the existing literature, identify key concepts, and determine the scope of research on a given topic, rather than to evaluate the methodological quality of individual studies.

Scoping reviews are designed to provide a comprehensive overview of the breadth of research rather than in-depth evaluations of study quality. This focus on breadth aligns with the aim of the scoping review to capture a wide range of literature and identify gaps in research, which is particularly important when exploring diverse and evolving fields (Levac et al., 2010).

Moreover, the research questions formulated for this review are concerned with the scope and nature of evidence related to risk factors, protective factors, and intervention strategies rather than the quality of the studies themselves. By including a broad spectrum of studies, the review seeks to offer an extensive overview of the evidence available without imposing restrictive criteria that could limit the inclusion of potentially relevant research [43].

Thus, the decision to forgo a risk of bias or quality assessment is in line with the methodological approach of scoping reviews, which prioritize inclusivity and comprehensiveness over critical

appraisal. This approach is supported by the framework and methodology outlined by Levac et al. (2010), which underscores the importance of capturing the breadth of research to inform future studies and identify areas requiring further investigation [44]". (**See page 12, method section, lines 291-309**).

4. DISCUSSION

4.1.Synthesis of findings

The discussion does not provide a detailed synthesis of the findings from the scoping review.

I strongly suggest that authors integrate specific findings from the review to illustrate key points. For instance, mention specific risk and protective factors identified and any intervention strategies that showed promise.

See. https://onlinelibrary.wiley.com/doi/book/10.1002/9780470693926

Author's Reply: Thank you for raising these important concerns. We have included the following in the discussion section (**See page 13-14, discussion section, lines 325-349**) ***:*** *"In our preliminary analyses, we identified several critical risk factors that significantly contribute to youth crime. Among these, substance use emerged as a predominant predictor, highlighting the role of addiction and substance abuse in escalating criminal behavior [8-10]. Lower academic achievement was also identified as a key risk factor, where youth with poor academic performance were more likely to engage in criminal activities, underscoring the importance of educational support and interventions [8-10]. Poor parental supervision was another significant factor, with inadequate parental oversight often leading to increased risk of youth involvement in crime [8-10]. Additionally, social disadvantage, including factors such as low income, unemployment, and living in disadvantaged neighborhoods, was strongly associated with higher levels of criminal behavior among youth [8-10]. To mitigate these risks, our analysis revealed two particularly effective protective interventions. The first is multisystemic*

therapy (MST), an evidence-based approach that targets the various interconnected factors contributing to delinquent behavior[11]. MST involves intensive interventions at the family and community levels, aiming to strengthen family dynamics, improve communication, and reduce the influence of negative peer groups. Studies consistently show that youth who undergo MST exhibit significantly lower levels of criminal activity [11]..

The second intervention is Multidimensional Treatment Foster Care (MTFC), which provides specialized foster care combined with comprehensive therapeutic interventions for at-risk youth [12]. MTFC is designed to address behavioral issues through structured support, including close supervision, consistent discipline, and positive reinforcement within a foster care setting. Research indicates that MTFC effectively reduces delinquent behavior and improves overall outcomes for youth involved in the program [12].

These findings suggest that addressing the identified risk factors through targeted interventions like MST and MTFC can play a crucial role in reducing youth crime and supporting at-risk individuals in developing healthier, more positive life trajectories [11, 12]".

Reviewer 4:

Response to Reviewer #4's Comments

Dear Reviewer,

We would like to express our gratitude for your positive feedback and thoughtful comments. Below is our response to each of your comments.

Comment 1:

This manuscript presents a well-organized and methodologically sound scoping review protocol. The authors have used established frameworks, such as the Arksey and O'Malley framework and PRISMA-ScR guidelines, which are appropriate for the study. While this is a protocol and does not yet include data or conclusions, the outlined plan is robust and should effectively guide the research process.

***Response*:**

Thank you for acknowledging the methodological soundness of our study and the appropriateness of using the Arksey and O'Malley framework and PRISMA-ScR guidelines. We have ensured that the protocol follows a rigorous structure that will facilitate the comprehensive mapping of literature on youth crime.

Comment 2:

Given that this is a scoping review protocol, statistical analysis is not conducted. The manuscript is focused on mapping existing literature, identifying gaps, and providing an overview of key concepts and evidence.

***Response*:**

We appreciate your understanding of the nature of the scoping review protocol, which aims to map existing literature and identify key gaps in research. We have clarified this objective throughout the manuscript and emphasized the importance of the scoping review approach in gathering broad insights into protective and risk factors related to youth crime.

Comment 3:

Since this is a protocol, no findings or data are presented yet. Once the scoping review is completed, the authors will need to ensure that all data underlying their results are made fully available in accordance with PLOS's data availability requirement.

***Response*:**

We fully understand the data availability requirements outlined by PLOS and will ensure that once the scoping review is completed, all data will be made publicly accessible through appropriate repositories, in accordance with journal policies. We have emphasized this commitment in the revised manuscript.

Comment 4:

The manuscript is clearly written, well-structured, and easy to follow. The authors have effectively communicated their research plan, and the language used is precise and professional.

***Response*:**

Thank you for your kind words regarding the clarity and structure of the manuscript. We have made efforts to maintain a high standard of clarity and precision throughout the manuscript and are grateful for your acknowledgment.

General Comments:

Overall, this manuscript outlines a clear and comprehensive protocol for conducting a scoping review on youth crime, with a particular emphasis on identifying key risk and protective factors as well as intervention strategies, especially for First Nations Populations. The methodology is appropriate, and the writing is clear.

***Response*:**

We appreciate your positive feedback on the clarity of our manuscript and the appropriateness of the methodology. We have carefully designed this protocol to ensure that it effectively captures the key risk and protective factors for youth crime, with particular attention to First Nations Populations.
