# Supplementary material for: Protective factors, risk factors, and intervention strategies in the prevention and reduction of crime among adolescents and young adults aged 12–24 years: A scoping review protocol
Source: PLoS One. 2024 Nov 19;19(11):e0312684. doi: 10.1371/journal.pone.0312684 (PMC11575785; doi:10.1371/journal.pone.0312684)
Supplement: Attachment — Submitted filename: ReviewerResponse_Letter (2).docx [file pone.0312684.s003.docx]

RESPONSE TO REVIEWERS

Professor Johannes John-Langba, Ph.D., M.P.H., M.S.WA

cademic Editor

PLOS ONE

Dear Professor Johannes John-Langba,

Thank you for your feedback on the manuscript submitted titled “Protective factors, risk factors, and intervention strategies in the prevention and reduction of crime in 12–24-year-old: A Scoping Review Protocol”. We appreciate the opportunity to revise our work. We would like to express our appreciation to the reviewers for their time and sharing their expertise to provide us with valuable suggestions and constructive comments. We have addressed all comments and suggestions within the manuscript. Please find below our responses to each comment raised.

Reviewer #1

|  | **Reviewer Comment** | **Page** | **Comment response and change details** |
| --- | --- | --- | --- |
| **1.** | Pages 1-3 are duplicated within the manuscript. Also, there is no clear Abstract, as the Abstract and Introduction are combined. | P2 | The pages have been checked to avoid duplication. The abstract has been updated to follow the unstructured abstract guidelines in PLOS One. |
| **2.** | On the bottom of page 6, last paragraph, there needs to be a subheading, "Aim of Study" or "Purpose of Study." | P4 | We have added the heading “Aims of Study” for the last paragraph as requested. |
| **3.** | Throughout the manuscript, there are both present and future tenses. If the study/review has already been conducted, it should be past tense. | All pages | The manuscript has been checked for the use of tense. We have used past tense up to the first paragraph of the method (as this explains what was done to create the protocol). Beyond this, future tense has been used to outline the intented steps of the scoping review. There are some exceptions as we added steps that were taken to create the protocol in past tense. |
| **4.** | There is no Findings section. What did you researchers find/conclude within the study/review? What do the Findings suggest in relationship to any proposed questions? | P8 | Given that the paper is a protocol, the proposed reporting of findings has been included on page 10 under the heading “Step 5: Collating, Summarising, and Reporting the Results”. |
| **5.** | The last paragraph discusses limitations, but what are the limitations based on? | P9 | This section has been re-worded to make it more clear that these limitation are some of the potential challenges that we may find during the review process and why these may be a limitation. |

Reviewer #2

|  | **Reviewer Comment** | **Page** | **Comment response/change details** |
| --- | --- | --- | --- |
| **Abstract** | | | |
| 1. | Authors to follow the guidelines on writing an unstructured abstract in PLOS One. | P1 | The abstract in the manuscript has been updated to follow the guidelines on writing an unstructured abstract in PLOS One have now been followed. |
| 2. | Provide a concise description of the main aim/objective of the scoping review and state this as an aim. E.g. The aim of this scoping review is… | P1 | A concise aim of the scoping review has been added:  “This article presents a scoping review protocol that aims to collate key risk/protective factors and intervention strategies in identifying youth at risk of crime.” |
| **Introduction** | | | |
| 3. | The introduction section can be synthesised into four succinct paragraphs. | P2-4 | The introduction section has been synthesised into the recommeneded four succinct paragraphs: introducing the main problem, the specific problem from a contextual perspective, the gap in the literature, and the review questionss. We have decided to include the justification of why the review questions and objectives lend themselved to a scoping review approach within the method. |
| **Methods** | | | |
| 4. | Authors to follow the PLOS One (PRISMA-ScR) Checklist on structuring the methods section. | P4-8 | The authors have maintained the structure of the methods to align with the five Arksey and O’Malley steps followed. We have also added any missing subheadings that align with the PLOS One (PRISMA-ScR) Checklist in this section and reorganised information within these. The additional sub-headings are: Information sources and search, data items, critical appraisal of individual sources of evidence.  Any other sub-headings from the checklist not added were covered with the headings of the Arksey and O’Malley steps. |
| 5. | Outline what the five steps of the Arksey and O’Malley framework are. | P4 | The five steps in the Arksey and O’Mally framework have been detailed in the manuscript:  “…and the Arksey and O’Malley framework [42],which follows five steps: (i) identifying the research question; (ii) identifying relevant studies; (iii) study selection; (iv) charting the data; and (v) collating, summarising, and reporting results.” |
| 6. | What framework was used to inform the development of the research questions? | P5 | The Arksey and O'Malley framework and JBI Manual were used to guide the formulation of research questions. We have added the following to clarify in the manuscript:  “It is recommended that research questions in a scoping review are broad and articulate the target population, concept and context [41, 42].” |
| 7. | Clarify if the data extraction process is informed by the Joanna Briggs Institute’s template for data extraction or which tool will be used for this. | P10 | We have added some information to clarify this:  “The data items recommended by the JBI Manual (Aromataris & Munn, 2020) and relevant to the research questions will be extracted from each included source and entered into a spreadsheet by one author.” |

Thank you for the feedback provided. We appreciate the opportunity to revise the manuscript and resubmit for further consideration.

Kind regards,

Rosanna Rooney

(SIGN)
