# Supplementary material for: Protective factors, risk factors, and intervention strategies in the prevention and reduction of crime among adolescents and young adults aged 12–24 years: A scoping review protocol
Source: PLoS One. 2024 Nov 19;19(11):e0312684. doi: 10.1371/journal.pone.0312684 (PMC11575785; doi:10.1371/journal.pone.0312684)
Supplement: Attachment — Submitted filename: Point-by-point response factors_v3. 08.10.24.docx [file pone.0312684.s006.docx]

**Point-by-point responses for comments**

## Dear Professor AKM Alamgir, PhD, Academic Editor, PLOS ONE

## We would like to thank you very much for giving us the opportunity to re-revise our manuscript entitled “*Protective factors, risk factors, and intervention Strategies in the prevention and reduction of crime among adolescents and young adults aged 12-24 years: A scoping review protocol*” [PONE-D-23-15234R2] - [EMID:1cee4b7f8c048fe8]. We would also like to express our thanks to the reviewers for sharing their expertise, detailed reviews, and providing us with valuable suggestions and constructive comments on our manuscript. We have fully addressed their comments in the revised manuscript. Following each comment and suggestion, the answer is provided together with references to the page and lines where the changes were made.

We believe changes have resulted in a significantly improved manuscript, which you will find uploaded alongside this document.

**Editor and Reviewers’ comments:**

**Editor’s comments:**

Please provide a point-by-point response to the reviewers' comments

***Response*:** *As requested, we have included a point-by-point response to all reviewer comments in this document.*

**Reviewer 3**

**General comments**

Dear Authors, I would like to commend you all for the effort in improving your review protocol manuscript and appreciate the opportunity to review your impeccable work.

***Response*: Thank you so much.**

Please see the minor revisions outlined below:

**Abstract:**

**• Conclusion** (Page 2, lines 63-65): Remove the sentence: “This scoping review protocol aims to systematically map key risk and protective factors, as well as intervention strategies, for preventing and reducing youth crime, including among First Nations populations.”

**Response:** This has been removed.

**• Conclusion (revision):** Remove the dash in “model—from” throughout the paragraph, as it does not reflect a professional writing style:

**Response:** This has been fixed now. The revised paragraph reads as follows: *“Anticipated findings suggest that current research has extensively examined factors across all levels of the socioecological model, from individual to community levels, revealing a predominant focus on individual-level predictors such as substance use, prior criminal history, and moral development. The review is expected to identify effective interventions that address critical factors within each domain, including Multisystemic Therapy (MST) and Multidimensional Treatment Foster Care (MTFC), which have shown promise in reducing youth crime. Additionally, it will likely highlight significant trends in risk and protective factors, such as the dual role of academic achievement—both as a risk and protective factor—and the impact of family-based interventions. The review will also address gaps in research, particularly regarding Indigenous youth, underscoring the need for targeted studies to better understand their unique challenges. These findings will guide future research and inform the development of comprehensive prevention and early intervention programs tailored to diverse youth populations.”***See pages 2-3, abstract section, lines 63-75):**

• Ensure that the abstract does not exceed 300 words, as per the journal's guidelines:

**Response:** Done.

**Introduction:**

• Page 5 (Introduction, line 129): Include the author’s surname in the sentence: “Critics argue that…”.

**Response:** Thank you for your suggestion. We have now revised the sentence as recommended: *Critics, such as Malvaso et al., argue that while these therapies can be beneficial, they may not adequately address broader socio-economic and systemic factors contributing to criminal behavior [27]. (***See page 5, introduction section, lines 127-129):**

**Critical Appraisal of Individual Sources of Evidence:**

• Revise the structure of the following sentence (Page 12, Line 291): “In accordance with the Arksey and O’Malley framework (2005) [30], the scoping review does not involve a formal risk of bias or quality assessment of included sources.”

**Response:** This has been fixed now. The revised sentence reads as follows: “*As suggested by the Arksey and O’Malley framework (2005) [30], this scoping review does not include a formal risk of bias or quality assessment for the included studies* (Arksey and O'Malley, 2005)”. **(See page 12, method section, lines 289-290).**

• Replace the term “Forgo” in the sentence on Page 12, line 305: “Thus, the decision to forgo a risk of bias….” with a more alternative term that will accommodate non-academic audience.

**Response:** Done. **(See page 7, method section, lines 303-304).**

• Adhere to the journal’s in-text referencing guidelines: “This approach is supported by the framework and methodology outlined by Levac et al. (2010)...”

**Response:** This has been fixed now.

PRISMA Flowchart:

• (Page 19, lines 475-476): In the flowchart, add a category for duplicates removed during the IDENTIFICATION phase to confirm whether additional records identified were included in the total screened records.

**Response:** **Done**
