# Supplementary material for: Protective factors, risk factors, and intervention strategies in the prevention and reduction of crime among adolescents and young adults aged 12–24 years: A scoping review protocol
Source: PLoS One. 2024 Nov 19;19(11):e0312684. doi: 10.1371/journal.pone.0312684 (PMC11575785; doi:10.1371/journal.pone.0312684)
Supplement: Attachment — Submitted filename: Reviewer comments PONE-D-23-15234.pdf [file pone.0312684.s002.pdf]

Reviewer comments: Protective factors, risk factors, and intervention strategies in the prevention and reduction of crime in 12–24-year-old: A Scoping Review Protocol

Identifying risk factors and developing risk typologies is an important area of study in crime prevention and reduction among youth. The authors have presented the gaps in the literature on at risk populations such as First Nations groups from countries such as Australia, Canada and New Zealand. As such, this paper presents the first scoping review of primary studies of protective factors, risk factors, and intervention strategies in the prevention and reduction of crime in 12-24-year-olds, inclusive of First Nations communities. The paper is well written and is addressing a very important topic, given that crime among young people as young as 12 has child protection implications and in general has social and economic consequences. However, paper has some important limitations especially regarding the method mentioned below.

Please find my comments following my review.

Abstract

1. Authors to follow the guidelines on writing an unstructured abstract in PLOS One
2. Provide a concise description of the main aim/objective of the scoping review and state this as an aim. E.g. The aim of this scoping review is…

Introduction

The introduction section can be synthesised into four succinct paragraphs

1. Paragraph one introduces the main problem
    i. Youth offending and why it is a problem?
    ii. What have other countries found about youth offending, risk factors and protective factors ?
    iii. How have other countries dealt with youth offending and how has the identification of protective and risk factors influenced youth offending interventions?
2. Paragraph two looks at the specific problem from a contextual perspective (i.e. among First Nations communities)
    i. What is the prevalence of youth offending among First Nations communities?
3. Paragraph three introduces the gap in the literature
4. Paragraph four explain/justify why the review questions/objectives lend themselves to a scoping review approach?

Methods

1. Authors to follow the PLOS One (PRISMA-ScR) Checklist on structuring the methods section
    a. Protocol and registration
    b. Eligibility criteria
    c. Information sources
    d. Search
    e. Selection of sources of evidence
    f. Data charting process
    g. Data items
    h. Critical appraisal of individual sources of evidence
    i. Synthesis of results
2. Outline what the five steps of the Arksey and O'Malley framework are
3. What framework was used to inform the development of the research questions?
4. Clarify if the data extraction process is informed by the Joanna Briggs Institute's template for data extraction or which tool will be used for this
